# Evaluation of two molecular detection platforms for gastroenteritis pathogens in treated sewage water in the Eastern province of Saudi Arabia

**DOI:** 10.1038/s41598-022-25702-4

**Published:** 2022-12-16

**Authors:** Fawaz A. Al-Wohaib, Ibtihaj Al-Sharif, Hassan Al-Zain, Donna Murad, Layla Al-Harbi, Maha Al-Mozaini

**Affiliations:** 1 Environmental Health Unit, Workplace Environment Division, Environmental Protection, Saudi Aramco, Al-Midra Tower, 9th floor, Dhahran, Saudi Arabia; 2 Immunocompromised Host Research Section, Department of Infection and Immunity, King Faisal Specialist Hospital and Research Centre, Riyadh, Saudi Arabia; 3 Department of Clinical Laboratories Sciences, College of Applied Medical Sciences, King Saud University, Riyadh, Saudi Arabia; 4 College of Medicine, Alfaisal University, Riyadh, Saudi Arabia

**Keywords:** Molecular biology, Environmental sciences

## Abstract

The ability to screen environmental water samples for gastroenteritis pathogens, particularly viruses remains challenging. Here, we investigated the presence of enteric viruses in treated sewage effluent water samples collected from a cooling tower in The Kingdom of Saudi Arabia (SA) from 2018 to 2019. Our ultimate aim was to determine the optimal handling and processing conditions for the water samples and the most sensitive detection method for the assessment of viral contamination. Sewage was collected before and after treatment at three defined zones. Samples were concentrated by ultracentrifugation and analyzed using a multiplexed bead-based assay system (Luminex technology) or multiplex PCR (QIAstat-Dx). The efficiency of these modalities to accurately detect virus contamination were subsequently compared. In total, 64 samples (16 controls and four treated samples per-control) were analyzed for 26 enteric pathogens. Of the samples, 98.7% were negative for viruses following treatment. Detection rates were higher for the multiplex PCR (QIAstat-Dx) system compared to the hybridization method, highlighting its higher sensitivity. The current water sewage treatment protocols in KSA could efficiently eradicate viral pathogens, minimizing their potential for waterborne transmission. We provide the first systematic analysis of two molecular detection methods for the assessment of gastroenteritis-associated pathogens from environmental samples in KSA. We conclude that the multiplex PCR (QIAstat-Dx) system outperforms the Luminex technology for the detection of virus pathogens in treated water samples.

## Introduction

Gastroenteritis-associated pathogens are transmitted via the fecal–oral route through contaminated water or untreated water sources such as sewage^1^. Enteroviruses belong to the Picornaviridae family and represent the smallest, non-enveloped viruses known to infect humans and animals. Enteroviruses can be isolated from ground, sea, sewage and fresh water environments and have caused several outbreaks of gastroenteritis across the globe^2^. Examples in the past 30 years include a number of outbreaks of hand, foot and mouth disease with associated neurological complications in the Asia–Pacific region and sporadic cases in Europe. Current regulations from the European Union include the detection of human enteroviruses as a parameter of water quality^3^. Many common wastewater treatment processes fail to completely inactivate these viruses rendering recreational waters vulnerable to viral contamination. The typical parameters assayed in water include microbiological infection via *Legionella pneumophila, Klebsiella pneumoniae* and total *coliforms.* Effective disinfection performance parameters are also frequently assayed including residual chlorine, pH and the temperature of the treated sewage effluent (TSE) feed.

Several high-throughput detection methods including ELISA and qRT-PCR-based amplification have been used to detect enteroviruses in water samples.^4^ Low levels of enteroviruses present in large bodies of water can establish infections in humans at low infectious doses, meaning rapid and sensitive detection technologies are required. qRT-PCR followed by sequencing is regarded as the most specific and sensitive method currently available^5^ and can be used to document the emergence of newly arising virus strains, monitor virus evolution and confirm the source of an outbreak^6,7^. This can however be limited by the presence of inhibitory components in collected samples that can lead to false-negative results. This poses a barrier and sample preparation for these assays remains time-consuming and laborious.

Several technologies have more recently emerged for more high-throughput detection of water borne pathogens. One example is the QIAstat-Dx Analyzer that uses a multiplexed real-time PCR system to simultaneously detect pathogen nucleic acids in biological samples and represents a closed system that contains all necessary reagents, enabling hands-off sample preparation. Using this system, detected real-time amplification signals can be automated using an integrated software platform and reported via an intuitive user interface to maximize throughput. The Luminex xMAP technology (x = analyte, MAP = multi-analyte profiling) also enables the rapid, cost-effective, and simultaneous analysis of multiple pathogens in a single sample. This methods involves the pre-coating of fluorescent microspheres with specific diagnostic antigens that capture the corresponding pathogens which are combined with fluorescent reporters recognized by the Luminex reader. This system can identify up to 500 targets in a single sample and has been utilized within multiple diagnostic studies.

Here, we performed the first systematic analysis of these two molecular methods to detect the presence of gastroenteritis-associated pathogens in treated water samples from a cooling tower in KSA. We show that current water sewage treatment protocols efficiently eradicate viral pathogens and recommend multiplex PCR (QIAstat-Dx) as the most sensitive detection technology.

## Materials and methods

### Treatment processes

Preliminary-, secondary- (Aeration and Clarifier Settlement) and tertiary treatment (sand filtration and disinfection) were performed as previously described^8^. Waste-water (WW) flowing from residential areas was collected in a junction box prior to entering the treatment facility. WW was delivered by haulage tankers and was disposed at the Septage receiving facility (SRF), which joins the influent pipeline to the head works. The head works was equipped with a preliminary treatment station to remove debris such as rocks and wooden materials. A grit chamber was used to remove heavier materials. The head works outlet chamber diverts the WW flow to emergency ponds which can be returned for treatment (Splitter Box #1) via the recycle wet-well. The total influent flow was measured using a parshall flume located in the channel before the outlet structure. The influent water quality was validated daily through the collection of samples and the assessment of dissolved oxygen (DO), pH, temperature, total suspended solids (TSS), Oil & Grease.

Secondary treatment processes are shown in Supplementary Fig. 1. WW from the head works flows to splitter box #1 and was split into tanks 1–2 for aeration. Splitter Box #1 also received a return sludge flow. The recycle wet well flowed from the continuous backwash sand filter. The emergency pond flowed as shown in Supplementary Fig. 1. Surface aerators maintained the required level of DO (2.0 mg/l) to ensure the microbiological degradation of organic matter. DO levels were validated on each shift and the surface aerator speed was manually adjusted. Shift samples were collected on a daily basis to validate the performance of the aeration tank. DO, pH, temperature, TSS, mixed liquor suspended solids (MLSS), mixed liquor volatile suspended solids (MLVSS), sludge retention times (SRT), sludge settleability and color, foaming or bulking were measured. The outlet from the aeration tanks was delivered to Splitter Box# 2 to clarifiers 1 and 2. The water quality of the clarifier outlet was validated through the assessment of turbidity, TSS, DO, pH and temperature. Operational factors including sludge blanket, torque indicators and water surface were noted each shift. Sludge collected from the clarifier was periodically discarded or returned to splitter box #1 based on operational parameters. Sludge waste from the clarifier was discharged to the aerobic digester for further degradation and disposal to the sludge drying beds. The return sludge was pumped to splitter box #1 to maintain biological activity in the aeration tank.

The tertiary treatment process is shown in Supplementary Fig. 3. Clarifier effluent water was continuously injected with a coagulant (Alum) to enhance the filtration process. Chlorine was added to the clarifier effluent line to prevent microbiological growth on the sand filter. The sand filter operated continuously with the WW and flowed into the wet well, where chlorine was added to inactivate microbiological contaminants. The chlorine contact time was 5 KM prior to TSE. EST pumps discharged the filtered water from the wet well to the effluent storage tanks.

Tertiary treated sewage effluent (TTSE) was analyzed by a third-party laboratory for compliance. Weekly analysis was performed for BOD, COD, TSS, total Coliform, intestinal eggs, pH, turbidity and temperature. Monthly sample analysis was performed for nitrate. Discharge from the effluent storage tanks was delivered to a 30″ header line for suction to community irrigation pumps.

### Sample collection and preparation

Water samples from the cooling tower were collected every other week in Dharan from April 14th, 2019 to December 3rd, 2019. Zones were classified as follows: Site A: control site with untreated sewage. Abqaiq STP post treatment (Irrigation pump discharge line); Site B: TSE water supply to the AC Plant # 2 Cooling Tower (CT feedline); Site C: AC Plant # 2 Cooling tower circulation line. Samples were aseptically collected in 1 l of sterile water in glass bottles and maintained at 4 °C in dark containers during transport to the King Faisal Specialist Hospital Research Center, Riyadh (KFSHRC). Samples were transported for ≤ 6 h prior to analysis.

A total of 64 samples were collected over a 9-month period. These included 16 pretreatment source/raw effluent (control) samples and 48 water-treated samples from each location. Samples were processed under controlled conditions within 12 h of arrival.

A two-step centrifugation method was used for the assessment of enteric viruses as previously described^12^. Samples were initially concentrated by ultracentrifugation at 20,450*g* for 24 h at 4 °C to 10 mL. A second round of ultracentrifugation was performed at 182,000*g* for 2 h at 4 °C SORVALL RC 6 PLUS (Adapter : SORVALL SLA-1500 SUPER_LITE). Samples were analyzed in parallel via multiplex PCR or hybridization for comparative screening and detection. Remaining samples were stored at 4 °C.

### Data collection

Samples were collected at Dharan and screened for: (1) microbiological parameters: *Legionella pneumophila, Klebsiella pneumoniae* and total *coliforms*; (2) disinfection parameters included residual chlorine, pH, temperature, physical and chemical analysis of the TSE feed and recirculating water.

### Molecular testing platforms

Concentrated water samples were analyzed using the QIAstat-Dx Gastrointestinal Panel (Qiagen, USA), a qualitative test designed for the detection of viral, parasitic or bacterial nucleic acids in stool samples. These included: *Vibrio vulnificus, Vibrio parahaemolyticus, Vibrio cholerae, Entamoeba histolytica, Cryptosporidium *spp.,* Giardia lamblia, Cyclospora cayetanensis, Campylobacter *spp.,* Enterotoxigenic E. coli *(*ETEC*),* Enteropathogenic E. coli *(*EPEC*),* Enteroaggregative E. coli *(*EAEC*), *Shiga-like toxin-producing E. coli *(*STEC *[*enterohemorrhagic E. coli*]),* Salmonella *spp.,* Clostridium difficile *(*tcdA/tcdB*)*, Yersinia enterocolitica, Shiga toxin-producing E. coli *(*STEC*)* serotype O157:H7, Enteroinvasive E. coli *(*EIEC*)*/Shigella, Plesiomonas shigelloides, Human Adenovirus F40/F41, Norovirus GI, Norovirus GII, Rotavirus A, Astrovirus and Sapovirus GI, GII, GIV* and *GV*. Concentrated water samples were loaded onto the gastrointestinal panel cartridge and nucleic acid extraction, amplification and detection were performed automatically on the QIAstat-Dx analyzer. RT-PCR amplification curves were subsequently generated.

The second molecular detection method used the Luminex platform, a multiplex amplification bead-based hybridization system based on the xTAG Gastrointestinal Pathogen Panel (GPP). Using a small sample volume, the system can simultaneously measure 100 analytes. Nucleic acid extraction and purification were performed using EZ1 Nucleic acid mini kit v2.0 (Qiagen, USA) as per the manufactures protocol. Purified nucleic acids were eluted in 60 μl of buffer. The xTAG Gastrointestinal Pathogen Panel detected an identical list of bacteria, viruses and parasites to the QIAstat-Dx Panel.

## Results

### Water sample preparation and analysis

A total of 64 water samples consisting of 16 controls and 48 test samples from the three filtration zones were analyzed. Samples were collected at the Dhahran site every 2 weeks. Physical and chemical parameters of each water sample were recorded. Average values are summarized in Table 1.

**Table 1:** Average physical and chemical performance parameters for samples collected at the Daharan site.

	Control sample sewage water	Test sample Zone A	Test sample Zone B	Test sample Zone C
Collection time	5:30	5:30	5:30	5:30
Delivery time	12:30	12:30	12:30	12:30
Volume (L)	1	1	1	1
Temperature °C (± SE)	NA	27.75	26.2	26.08
pH (± SE)	NA	7.9	7.86	8.06
Turbidity (%)	NA	100	100	70.58
Odor (%)	NA	100	100	100
Free chlorine residual	NA	5.594	4.534	3.271
TDS/conductivity	NA	0.686	0.68	0.66

*NA not applicable.

Peak virus recovery was observed following two rounds of ultracentrifugation. Sewage samples (n = 16) were included as positive controls. Concentration of the water samples led to recovery rates of 59.34% on the QIAstat-Dx platform compared to 6.2% on the Luminex 200 platform, highlighting its superior performance for analysis (Table 2).

**Table 2:** Virus recovery in sewage following ultracentrifugation.

Sample ID	Norovirus GII		Norovirus GI		Astrovirus		Sapovirus		Adenovirus F40/F41		Rotavirus A	
	QIAstat	Luminex	QIAstat	Luminex	QIAstat	Luminex	QIAstat	Luminex	QIAstat	Luminex	QIAstat	Luminex
Control **1**	** + **	**−**	** + **	**−**	** + **	**−**	** + **	**−**	** + **	** + **	**−**	**−**
Control **3**	** + **	**−**	** + **	** + **	** + **	**−**	** + **	**−**	**−**	**−**	**−**	**−**
Control **4**		** + **	** + **	**-**	** + **	**−**	** + **	**−**	** + **	**−**	**−**	**−**
Control **5**	** + **	**-**	** + **	**-**	** + **	**−**	** + **	**−**	** + **	**−**	**−**	**−**
Control **6**	** + **	**-**	** + **	**-**	** + **	**−**	**−**	**−**	** + **	**−**	** + **	**−**
Control **7**	** + **	**−**	**−**	**−**	** + **	**−**	** + **	**−**	**−**	**−**	** + **	**−**
Control **8**	**−**	**−**	**−**	**−**	** + **	**−**	**−**	**−**	**−**	**−**	** + **	**−**
Control **9**	**−**	**−**	**−**	**−**	**−**	**−**	** + **	**−**	** + **	**−**	**−**	**−**
Control **10**	** + **	**−**	**−**	**−**	** + **	**−**	** + **	**−**	** + **	**−**	** + **	**−**
Control **11**	**−**	**−**	** + **	**-**	** + **	**−**	** + **	**−**	** + **	**−**	**−**	**−**
Control **12**	** + **	**−**	** + **	** + **	** + **	**−**	** + **	**−**	** + **	**−**	**−**	**−**
Control **13**	**−**	** + **	** + **	**−**	** + **	**−**	** + **	**−**	** + **	**−**	** + **	**−**
Control **14**	**−**	**−**	**−**	**−**	** + **	**−**	** + **	**−**	** + **	**−**	**−**	**−**
Control **15**	** + **	**−**	** + **	**−**	** + **	**−**	** + **	**−**	** + **	**−**	**−**	**−**
Control **16**	**−**	**−**	**−**	**−**	** + **	**−**	**−**	**−**	** + **	**−**	**−**	**−**

### Molecular analysis of water samples

**Figure 1:**
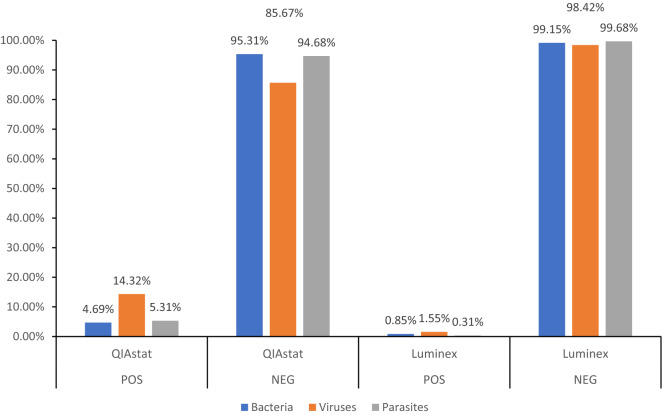
Detection of water borne pathogens using the QIAstat-Dx vs. Luminex 200 platforms. Concentrated water samples were loaded onto the gastrointestinal panel cartridge of each platform. Nucleic acid extraction, amplification and detection were performed automatically on the QIA-stat-Dx platform or using an EZ1 Nucleic acid mini kit v2.0 for the xTAG Gastrointestinal Pathogen Panel. Of the 64 samples, the QIA-stat-Dx platform detected positive rates of 4.69%,14.32%, 5.31% and negative rates of 95.31%, 85.67%, and 94.68% respectively for bacteria (blue bars), viruses (orange bars) and parasites (grey bars). The Luminex platform detected positive rates of 0.85%, 1.55%, 0.31% and negative rates of 99.15%, 98.42%, and 99.68% respectively for bacteria, viruses and parasites. POS: total number of positive samples vs. NEG: total number of negative samples. Percentages indicate the number of samples positive for a specific pathogen.

We evaluated the performance of the QIAstat-Dx and Luminex 200 platforms to detect bacteria, viruses and parasites from both test and control samples (Fig. 1). In control sewage, both platforms could detect viruses, bacteria and parasites to a sensitivity ≥ 95% (Fig. 1). Upon analysis of the test samples, the QIAstat-Dx could identify at least one gastroenteritis-associated pathogen in samples obtained from the three zones, with detection rates of 4.69% for bacteria, 14.32% for viruses and 5.31% for parasites. No enteric pathogens were detected in the remaining 48 samples. By comparison, the Luminex 200 platform detected gastroenteritis-associated pathogens in 11 water samples from the three zones, with detection rates of 0.85% for viruses, 1.55% for bacteria and 0.31% for parasites. No enteric pathogens were detected in the remaining 53 samples (Fig. 1). Virus detection using the QIAstat-Dx platform (14.32% vs 0.85%) was therefore more sensitive than the Luminex 200 analysis.

The performance and concordance of each platform are summarized in Table 3. A concordance of 27% was observed for *E-Coli* (st/it) (3/13), 50% for Norovirus GI (4/8), and 20% for Adenovirus F40/F41 (2/10). *Clostridium difficile* toxin A/B was detected only on the Luminex 200 platform (1 sample in 64). Positive detection was observed in 2 treated samples but no gastroenteric pathogens were detected. Testing was repeated on the Luminex 200 platform for confirmation. Discordant results were observed for 19 targets (QIAstat-/Luminex+, QIAstat+/Luminex−). The QIAstat-Dx platform detected 12 gastroenteritis pathogens that were not detected on the Luminex 200 platform, including *Yersinia Enterocolitica, Enteropathogenic E-coli, Enteroaggregative E. Coli, Vibro Cholera, Entroinvasive E.coli/shigella, Sapovirus, Rotavirus A, Astrovirus, Norovirus GII, Giardia Lambia, Entamoeba Histolytica,* and *campylobacter *spp. Five targets (*Vibrio Parahaemolyticus, Vibrio Vulnificus, Plesiomonas Shigelloides, Cyclospora cayetanensis, STEC O157* showed low detection rates on both platforms.

**Table 3:** Comparison of the detection rates of Enteric pathogens between the QIAstat-Dx and Luminex 200 platforms.

Target	QIAstat^+^/Luminex^+^	QIAstat^+^/Luminex^-^	QIAstat^-^/Luminex^+^	QIAstat^-^/Luminex^-^
**Parasite**				
*Cryptosporidium *spp.	0	4	1	59
*Cyclospora cayetanensis*	0	0	0	64
*Giardia Lambia*	0	7	0	57
*Entamoeba Histolytica*	0	2	0	62
*campylobacter *spp.	0	3	0	61
**Bacteria**				
*Vibrio Parahaemolyticus*	0	0	0	64
*Vibrio Vulnificus*	0	0	0	64
*Yersinia Enterocolitica*	0	1	0	63
*Enteropathogenic E-coli*	0	7	0	57
*Plesiomonas Shigelloides*	0	0	0	64
*STEC*	0	2	2	60
*STEC O157:H7*	0	0	0	64
*E-Coli (st/it)*	3	8	0	53
*Salmonella*	0	1	1	62
*Enteroaggregative E.Coli*	0	9	0	52
*C.Difficili Toxin A/B*	0	0	1	63
*Vibro Cholera*	0	5	0	59
*Entroinvasive, E.coli/shigella*	0	3	0	61
**Viruses**				
*Norovirus GII*	1	10	0	53
*Norovirus GI*	1	7	2	53
*Astrovirus*	0	14	0	50
*Sapovirus*	0	9	0	55
*Adenovirus F40/F41*	2	7	0	55
*Rotavirus A*	0	4	0	60

## Discussion

Cooling systems, particularly open water systems, support the growth of many microorganisms and biofilms. If uncontrolled, these lead to serious adverse effects. Contamination can be controlled using optimized water treatment programs consisting of effective microbiological monitoring, testing and control strategies. Here, we performed a comprehensive analysis of two advanced platforms for the detection of microorganisms including enteroviruses. Our analyses showed that the QIAstat-Dx platform is threefold more effective than the Luminex 200 for the detection of water borne pathogens and that current disinfection procedures in KSA appear effective in the removal of these microorganisms. We therefore provide the first systematic analysis of these two molecular detection methods in treated water samples in KSA.

Disease outbreaks from cooling towers have led to fatalities, primarily due to inadequate water and chemical treatment. *Klebsiella *sp. are natural inhabitants of sanitary water systems that can multiply under nutrient rich conditions. Their ingestion or transmission through contaminated aerosols can lead to pneumonia, particularly in immune-compromised individuals. Wastewater treatment plans can be designed for pathogenic human viruses, though these are dependent on the geographical area and type of virus circulating in the population. Although primary and secondary treatment processes can reduce virus titers, they are not specifically designed for this purpose. Tertiary treatments including filtration, membrane technology and UV-light systems are often employed as a multiple barrier approach. The risk to populations from virus-bearing aerosols are currently theoretical, with no published outbreak reported. Furthermore, viruses will not replicate outside of their host and their fate is dependent on a range of environmental factors including temperature, humidity and sunlight. It is however recognized that enteric viruses, including Rotavirus and Adenoviruses pose a risk to human health during the irrigation of landscapes in unrestricted areas. A key to effective monitoring is confidence in the isolation procedures used to study water borne pathogens. We observed peak virus recovery in all water samples assessed following two rounds of ultracentrifugation. Concentration of the water samples led to recovery rates of 59.34% on the QIAstat-Dx platform compared to 6.2% on the Luminex 200 platform, again highlighting its superior performance. We therefore highlight both the optimal isolation and analysis procedures to maximize virus recovery and ensure accurate sample analysis.

The use of primary biocides such as sodium hypochlorite, a popular disinfection method, fail to effectively remove viruses from treated effluent in isolation, with multiple barrier approaches often required to effectively control the risk. Cooling systems provide an environment where micro-organisms such as protozoa, algae, fungi and bacteria including legionella and klebsiella can proliferate. Human exposure through bio-aerosols relates to ‘drift’ from the integral drift eliminators, ‘windage’ directly from the cooling tower basin and fugitive exposure due to structural leakage. When water temperatures range from 20 to 50 °C (68°–122° F), cooling towers have the potential for microbiological growth. Water treatment, operational control and maintenance practices are therefore required. Even with an appropriate design, water droplets small enough to be inhaled (i.e. < 5 μm in diameter) can leave the drift eliminator^9–11^. The outcome of one pilot study indicated that TSE is a viable alternative to groundwater for industrial cooling systems with potential worldwide use. Microbiological growth in the cooling tower can be controlled by the continuous disinfection of recirculating water using 12.5% sodium hypochlorite as a primary biocide. Comprehensive water treatment systems, including robust operating procedures, advanced virus-testing technologies and ongoing governance and monitoring by a qualified specialist are essential for the management and control of adverse risks to human health. Determining the effectiveness of disinfection and non-oxidizing biocide treatments are required to enable the widespread use of TSEs in Cooling Tower Systems. Our analysis provides confidence in current disinfection procedures in KSA for the removal of such pathogens.

## Conclusions

This pilot study provides the first systematic analysis of two molecular detection methods to assess the presence of gastroenteritis-associated pathogens in treated water samples from cooling towers. We show that current water sewage treatment protocols can efficiently eradicate viral pathogens and recommend multiplex PCR (QIAstat-Dx) as the most sensitive detection technology.

## Supplementary Information

Supplementary Figures.

## Data Availability

All data generated or analyzed during this study are included in this published article [and its supplementary information files].
