# Supplementary material for: Evaluation of two molecular detection platforms for gastroenteritis pathogens in treated sewage water in the Eastern province of Saudi Arabia
Source: Sci Rep. 2022 Dec 16;12:21744. doi: 10.1038/s41598-022-25702-4 (PMC9758226; doi:10.1038/s41598-022-25702-4)
Supplement: Supplementary file 1 — Supplementary Figures. [file 41598_2022_25702_MOESM1_ESM.docx]

Article

**The** **evaluation of two different molecular detection platforms for gastroenteritis pathogens in treated sewage water in the Eastern province of Saudi Arabia.**

Fawaz A. Al-Wohaib^1^, Ibtehaj Al-Sharif^3^, Hassan Al-Zain^2^, Layla Al-Harbi^3^ and Maha Al-Mozaini^3^*

^1^ Environmental Health Unit, Health Protection Division, Environmental Protection, Saudi Aramco, Al-Midra Tower, 9th Floor, Dhahran, Saudi [Arabia. fawaz.alwohaib@aramco.com](mailto:Arabia.%20fawaz.alwohaib@aramco.com)

^2^  Environmental Science, Sustainability and Policy Group, Green Energy and Environmental Policy Department, Environmental Protection, Saudi Aramco, Al-Midra Tower, 9th Floor, Dhahran, Saudi Arabia. Hassan.alzain@aramco.com

^3^ Immunocompromised Host Research Section, Department of Infection and Immunity, King Faisal Specialist Hospital and Research Centre, Riyadh, Saudi Arabia [mmozaini@kfshrc.edu.sa](mailto:mmozaini@kfshrc.edu.sa) , ialsharif@kfshrc.edu.sa , [f1515212@kfshrc.edu.sa](mailto:f1515212@kfshrc.edu.sa).

***** Correspondence: mmozaini@kfshrc.edu.sa

**Supplementary Figure 1**


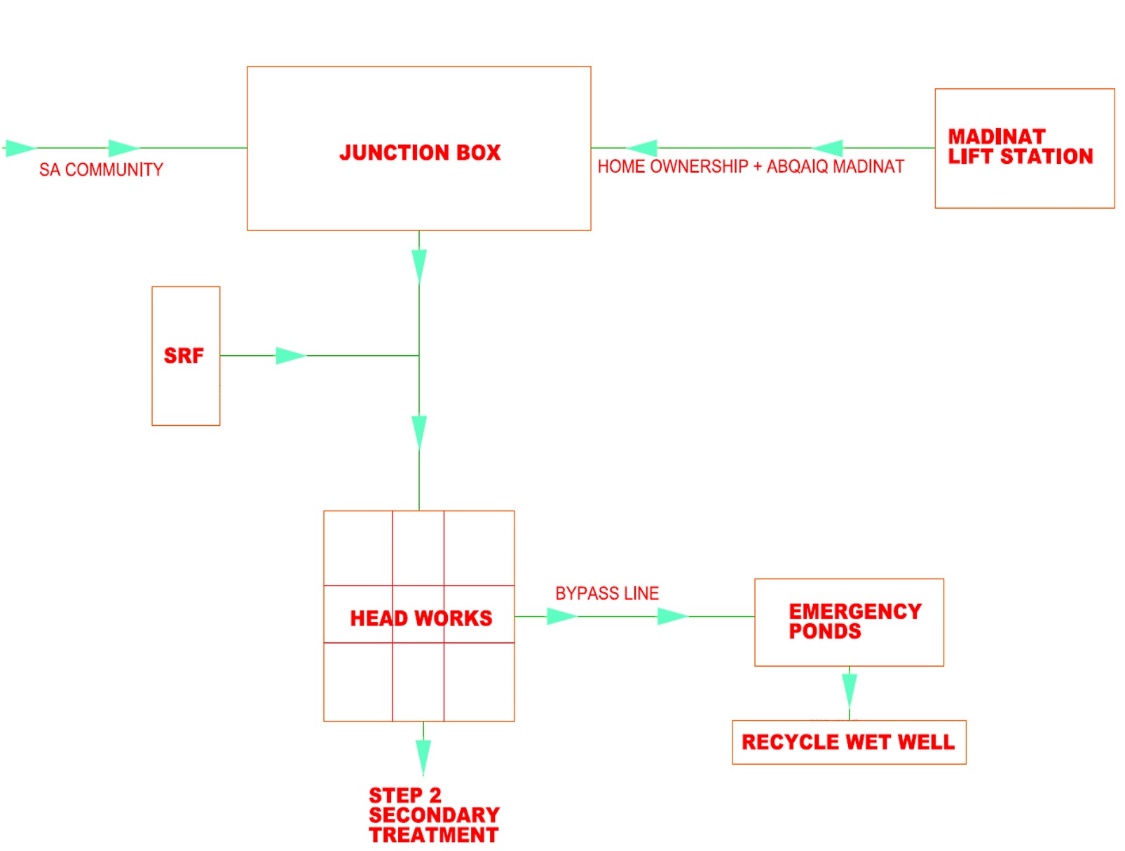


**Residential Area**

**Figure 1. Preliminary Treatment**. the facility uses the following wastewater treatment steps to achieve the Tertiary treated sewage effluent (TSE) quality. WW flows from Residential areas is collected in junction box before entering treatment facility and flows to head works. WW delivered by hauling tankers is disposed at Septage Receiving facility (SRF) which joins the influent pipeline before the head works as shown. The head works provides preliminary treatment using bar racks to remove debris (rags, rock material, wooden material. etc.) and grit chamber that allows settlement of heavier material. The Head works outlet chamber has provision to divert WW flow to emergency ponds which can be returned at a later stage to the main process treatment (Splitter Box #1) via the recycle wet well. Total influent flow received at the facility is measured by Parshall flume located in the channel before the outlet structure in head works. Influent water quality is validated during the daily shift by collecting samples for dissolved oxygen, pH, temperature, Total Suspended solids (TSS), Oil & Grease.

**Supplementary Figure 2**


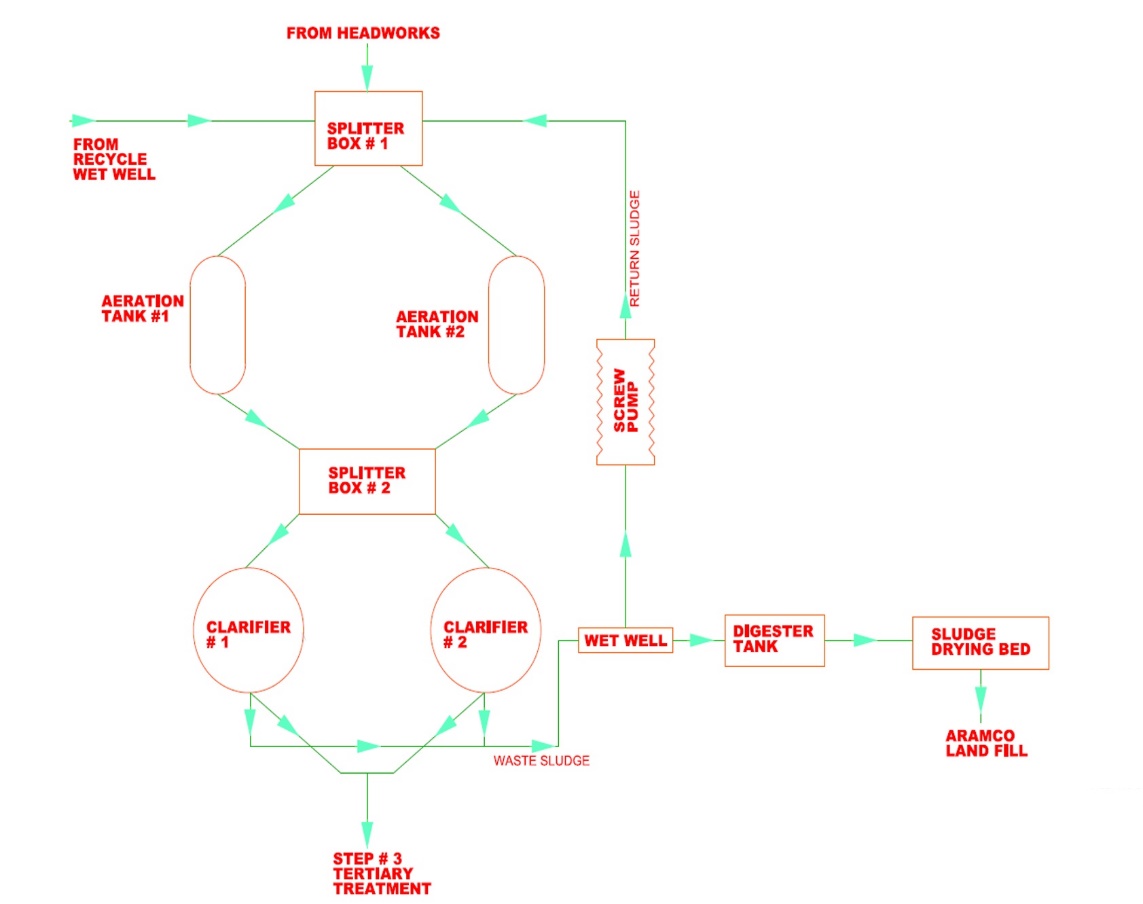


A

B


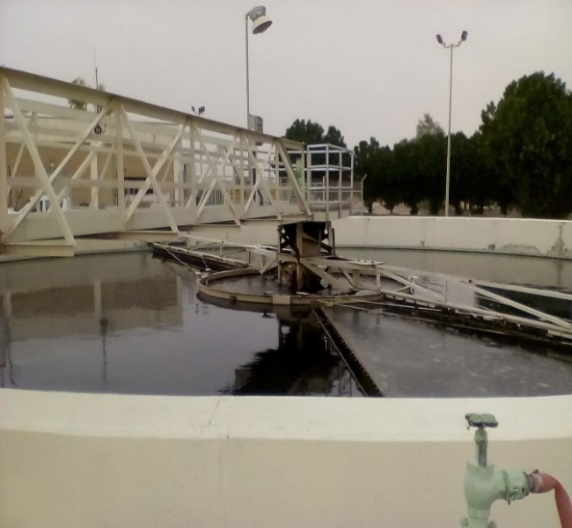

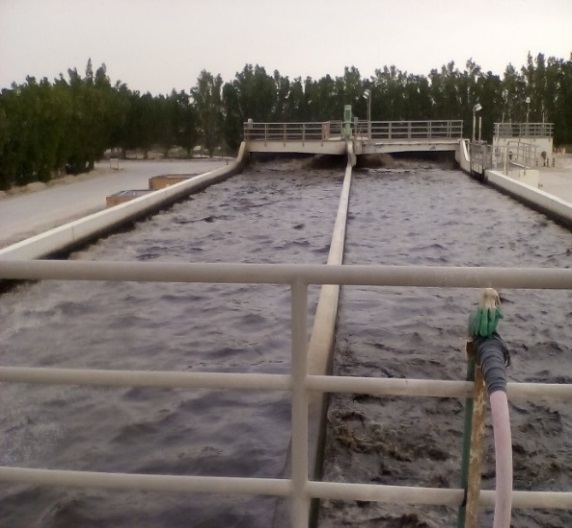


|  |
| --- |

**Figure 2. Secondary Treatment.** where the (A) Preliminary treated WW from head works flows to splitter box #1, where it is equally split for the aeration tanks 1 & 2. Splitter Box #1 also receives return sludge flow and recycle wet well flows from continuous backwash sand filter and emergency pond flows as (B). Surface aerators in aeration tank operate to maintain the required level of dissolved oxygen (2.0 mg/l) for microbiological degradation of organic matter. The DO level is validated every shift and surface aerator speed adjusted manually as required. Daily shift samples are collected to validate the aeration tank process performance for dissolved oxygen, pH, temperature, Total Suspended Solids (TSS), Mixed Liquor Suspended Solids (MLSS), Mixed Liquor Volatile Suspended Solids (MLVSS), Sludge Retention Time (SRT), Sludge settleability and aeration tank visual observation for color, foaming or bulking. The outlet from aeration tanks is delivered to Splitter Box# 2 where it equally splits the flow to Clarifiers 1 & 2. The clarifier outlet water quality is validated every shift by checking turbidity, TSS, DO, pH and temperature. Operational factors such as sludge blanket, torque indicator and water surface visual observations are noted every shift. The sludge collected from clarifier bottom is periodically wasted or returned to splitter box #1 based on the operational parameters (DO, turbidity, MLSS, Settle ability, Sludge blanket thickness and microbiological conditions). The wasted sludge from the clarifier is controlled and discharged to the aerobic digester for further degradation and disposal to sludge drying beds. The return sludge is pumped to splitter box #1 to maintain the biological activity in the aeration tank.

**Supplementary Figure 3**


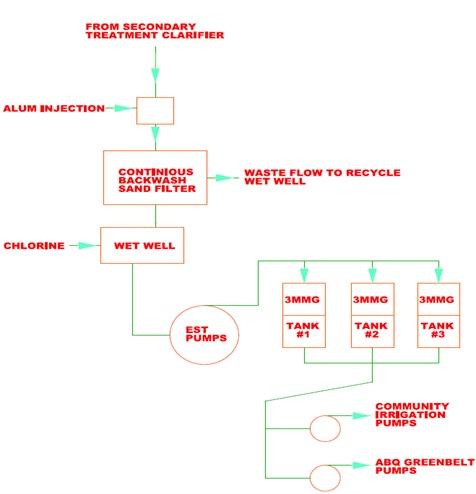


**Residential Area**

A

B


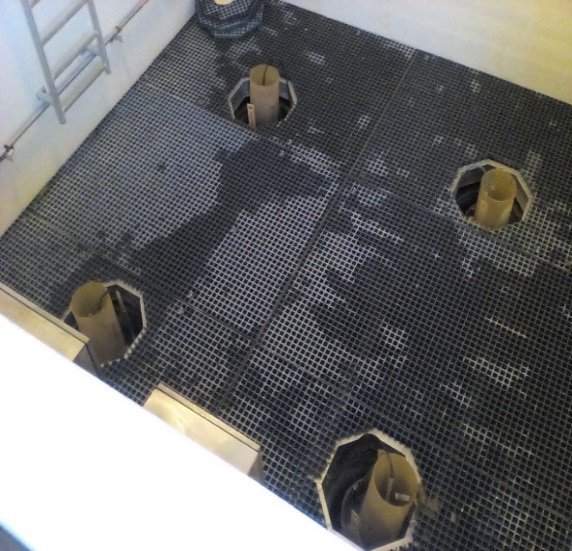

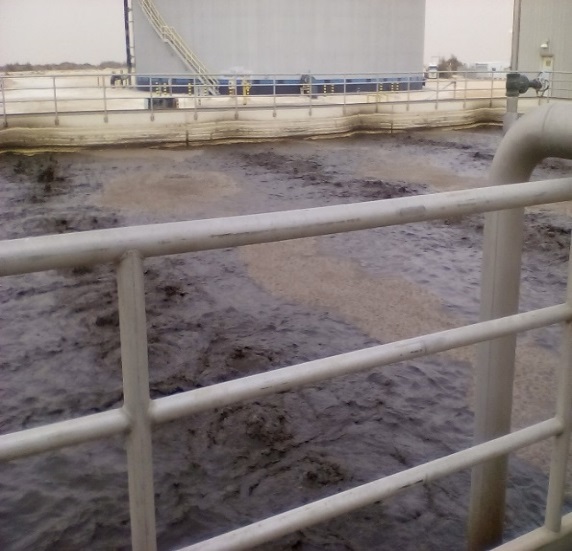


**Figure 3. Tertiary Treatment (Sand Filtration and Disinfection)**. The tertiary treatment process is shown (A) and (B). Clarifier effluent water is continuously injected with a coagulant (Alum) to enhance the sand filtration process. Chlorine is added to clarifier effluent line to prevent microbiological growth on the sand filter media. Sand filter operates continuously with the WW moving up through the sand filter media and the sand media continuously moving downward. Sand filter effluent flows into a wet well, where chlorine is added to achieve inactivation of microbiological and enteric virus parameters. The chlorine contact time is achieved in the wet well, the effluent storage tanks and approximately 5 KMs of transmission pipeline before TSE is utilized. EST pumps discharge the filtered water from the wet well to the effluent storage tanks 1, 2 &3. The tertiary treated sewage effluent (TTSE) is analyzed by third party laboratory for compliance parameters on weekly and monthly basis. Weekly analysis is for BOD, COD, TSS, Total Coliform, Intestinal eggs, pH, turbidity and temperature. Monthly sample is for nitrate. The discharge from the effluent storage tanks is delivered to a 30” header line for suction to Community Irrigation pumps.
